# Parents’ perceptions on offspring risk and prevention of anxiety and depression: a qualitative study

**DOI:** 10.1186/2050-7283-2-17

**Published:** 2014-06-30

**Authors:** Helma Festen, Karen Schipper, Sybolt O de Vries, Catrien G Reichart, Tineke A Abma, Maaike H Nauta

**Affiliations:** Department of Clinical Psychology and Experimental Psychopathology, University of Groningen, Grote Kruisstraat 2/1, Groningen, 9712 TS The Netherlands; Department of Medical Humanities, VU University Medical Center, Postbus 7057, Amsterdam, 1081 BT The Netherlands; Mental Health Care Friesland (GGz Friesland), Borniastraat 34B, Leeuwarden, 8934 AD The Netherlands; Curium-LUMC, Leiden University Medical Center, Leiden, The Netherlands; EMGO + Institute for Health and Care Research, VU University Medical Center, Van der Boechorststraat 7, Amsterdam, 1081 BT The Netherlands; Child and Adolescent Psychiatry, University Medical Center Groningen, Hanzeplein 1, Groningen, 9713 GZ The Netherlands

**Keywords:** Prevention, Offspring, Anxiety, Depression, Parent, Participation, Qualitative Research

## Abstract

**Background:**

Offspring of patients with anxiety or depression are at high risk for developing anxiety or depression. Despite the positive findings regarding effectiveness of prevention programs, recruitment for prevention activities and trials is notoriously difficult. Our randomized controlled prevention trial was terminated due to lack of patient inclusion. Research on mentally-ill parents’ perceptions of offspring’s risk and need for preventive intervention may shed light on this issue, and may enhance family participation in prevention activities and trials.

**Methods:**

Qualitative data were collected through semi-structured interviews with 24 parents (patients with anxiety or depression, or their partners). An inductive content analysis of the data was performed. Five research questions were investigated regarding parents’ perceptions of anxiety, depression, and offspring risk; anxiety, depression, and parenting; the need for offspring intervention and prevention; and barriers to and experiences with participation in preventive research.

**Results:**

Parental perceptions of the impact of parental anxiety and depression on offspring greatly differed. Parents articulated concerns about children’s symptomatology, however, most parents did not perceive a direct link between parent symptoms and offspring quality of life. They experienced an influence of parental symptoms on family quality of life, but chose not to discuss that with their children in order to protect them. Parents were not well aware of the possibilities regarding professional help for offspring and preferred parent-focused rather than offspring-focused interventions such as parent psycho-education. Important barriers to participation in preventive research included parental overburden, shame and stigma, and perceived lack of necessity for intervention.

**Conclusions:**

This study highlights the importance of educating parents in adult health care. Providing psycho-education regarding offspring risk, communication in the family, and parenting in order to increase parental knowledge and parent–child communication, and decrease guilt and shame are important first steps in motivating parents to participate in preventive treatment.

**Electronic supplementary material:**

The online version of this article (doi:10.1186/2050-7283-2-17) contains supplementary material, which is available to authorized users.

#15 *“It is not a question whether they [the children] will be affected, but rather what the effects will be.”*

## Background

Anxiety and depressive disorders are highly prevalent, and often co-occur, posing a huge burden on patients (Whiteford et al. 2013; De Graaf et al. 2012). Parental anxiety and depression present a significant threat to the mental health of their offspring. Children of anxious and depressed parents are at 3 – 4 times greater risk for developing these and other psychiatric disorders than children in the general population (Lieb et al. 2002; England & Sim 2009; Micco et al. 2009). Therefore, developing and testing the efficacy of interventions to prevent adverse outcomes in this population is of utmost importance.

To the best of our knowledge, five randomized controlled trials have specifically focused on preventing psychopathology in offspring of patients with anxiety (Ginsburg 2009) or depressive disorders (Beardslee et al. 2007; Clarke et al. 2001; Compas et al. 2009). Results of these prevention interventions seem positive, with interventions decreasing the risk for anxiety and depression by 41% (relative risk = 0.59) (Siegenthaler et al. 2012). Looking in more detail at the recruitment phase, however, targeted interventions seem to be subject to an important limitation: many contacted parents and offspring decline participation. For example, in the sample used by (Clarke et al. 2001), the 94 adolescent offspring (aged 13–18) of adults treated for depression were derived from an initial sample of nearly 3000 parents and 3400 youth, of which 2250 families actively declined participation.

**Figure 1:**
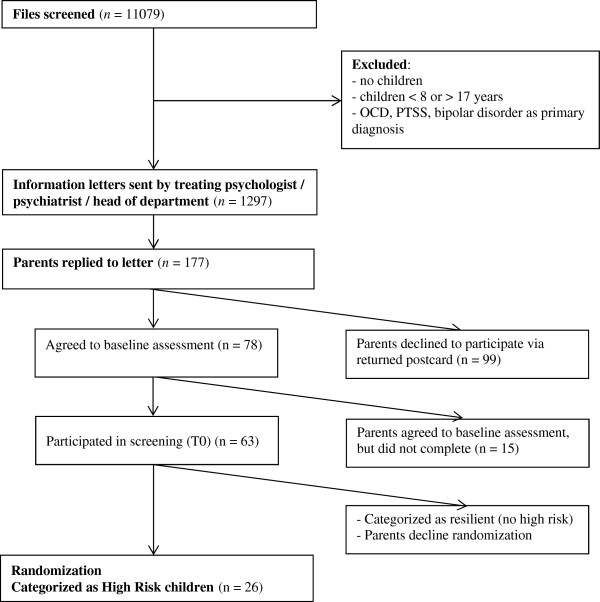
**Flowchart of inclusion and attrition of participants of prevention study (Nauta et al.** 2012 **).**

To contribute to this body of research, our own group also designed a prevention study for offspring, consisting of a screening and a randomized controlled trial. Notably, this study took the following features into consideration: first, that anxiety and depression are highly comorbid (Kessler et al. 2005) and, second, that intergenerational transmission of disorders is non-specific (Micco et al. 2009; Beidel & Turner 1997). The study thus targeted both anxiety and depression and was designed for offspring with current subdiagnostic levels of anxiety and depressive symptoms. It assessed additional risk factors, in order to select a high risk group to enhance treatment impact (Nauta et al. 2012).However, recruitment difficulties prevented completion of the original project. Across a period of 30 months, although we managed to screen as many as 11079 files, and sent 1297 information letters to families, only 78 positive reactions were obtained; recruitment was only 6% of the planned sample. Subsequently, we conducted baseline assessments and screening on 63 children and eventually we were only able to randomize 26 high risk children (of the 204 needed for sufficient power). This small sample size finally led to cancellation of the RCT. For inclusion and attrition of participants see Figure 1.

It became evident to us that this specific group of parents and offspring is generally reluctant or hesitant to participate in preventive research. In order to enhance participation in prevention activities, we reasoned that it would be important to obtain more insight into the perspectives of the target group. Qualitative studies are suitable for exploring the actual experiences and perceptions of patients, since they allow scope for patients’ narratives without being constrained by specific hypotheses of the researcher, and without being led by questionnaires and predefined items. Especially when little is known about the researched phenomenon, as in this case, qualitative research seems most appropriate.

In fact, qualitative studies have contributed to the understanding of various clinical issues, including patients’ needs and wishes (Kuper et al. 2008). Using qualitative methods to study the perspectives of patients may even suggest new themes that are overlooked in quantitative research (Schipper et al. 2013). The perspectives of the parents are of importance when one is conducting prevention research with children: they are pivotal in the process of evaluating the needs of their children. Furthermore, knowledge about the perspectives and needs of the mentally ill parent and the partner will be crucial in the decision making on participation in prevention activities.

What studies are available on this topic in the scientific literature? Research on patients’ perspectives on prevention for their offspring (i.e., with regard to anxiety and depression) is limited. Only a few qualitative studies have specifically reported on mentally ill parents’ barriers to participation in offspring intervention. Boyd, Diamond, and Bourjolly (Boyd et al. 2006) conducted focus groups with 18 mothers with depressive disorders or schizophrenia, discussing depressive symptoms, generational legacy, parenting difficulties, child problems, social support, stressful life events, therapy and other helpful activities, and desired (preventive) family treatment. They concluded that barriers for mothers in attending such an intervention included time constraints with school schedules, older children refusing to participate, embarrassment, and juggling with many demands. However, these barriers mostly related to the stressful life conditions and limited resources of low income, urban, ethnic minority, single mothers in the sample. It remains unclear whether these barriers hold for depressed parents in general.

Another study by Stallard et al. (Stallard et al. 2004) was not specifically on depressed mothers, but included 24 parents with a variety of severe mental disorders (psychosis, schizophrenia, bipolar disorder, and personality disorders). In this study, major problems in recruitment were also reported: for example, an initial parent–child group training was abandoned due to a lack of referrals. Parents themselves reported three main barriers to taking part in the preventive research. First, parents’ own needs for treating their mental health problems overwhelmed and obscured the needs of their children. Second, the responses of a number of parents suggested that they did not recognize or acknowledge the possible adverse effects of their illness on their children. And last, parents wanted to protect their children from further distress.

These two studies, however, lack information that may be relevant in the context of prevention activities. First, they only included patients and did not encompass partners’ perspectives. Also, the studies included broader samples of patients with a range of axis 1 and 2 disorders instead of a more specific group with possibly specific needs. Secondly, previous studies have not addressed parents’ perceptions of the need for professional help for offspring, or attention to this topic in parent treatment. Finally, barriers to optimal child (preventive) treatment have only been addressed in general terms; previous studies have not directly addressed barriers to participation in a randomized controlled trial. The latter seems critical: recruitment barriers appear to be common in randomized controlled trials (Ross et al. 1999) and may give rise to specificity of the collected sample (selection bias), affecting the validity and increasing the risk of not reaching a specific group of participants.

In sum, offspring of anxious and depressed parents are at high risk for developing these disorders themselves. Therefore, there is an urgent need for preventive research. However, recruitment of an adequate number of research participants has proven to be challenging. As parents are central figures in obtaining access to their children, insight in parents’ perspectives is important. It has been theorized that before investigating parents’ reasons for (not) participating in a study on offspring risk for anxiety and depression, parents’ perceptions of offspring risk and resilience, and whether parents’ link their own psychopathology and parenting style to offspring risk should be first investigated. Therefore, the current study uses qualitative interviews with a broad group of parents (patients and partners, mothers and fathers, with anxiety and depression, with several levels of severity) to investigate the following research questions:

1. What are parents’ (fathers and mothers, patients and partners) experiences with regard to their own depressive and anxiety disorders and their children’s vulnerability and resilience?
2. What are parents’ experiences with regard to their own depressive and anxiety disorders and parenting?
3. What are parents’ experiences with professional help and is there a perceived need for professional help (e.g. preventive interventions) for their children?
4. What are parents’ reasons for (not) participating in a prevention study (screening and a randomized controlled trial) with their children?
5. What are parents’ experiences with regard to participating in a prevention study?

## Methods

### Ethics statement

This research was a qualitative sub-study nested within a larger prevention study, consisting of screening of offspring for additional risk factors and a randomized controlled trial (STERK-study, Screening and Training: Enhancing Resilience in Kids; approved by the Medical Ethics Committee of the University Medical Center Groningen, NTR2888) (Nauta et al. 2012). Both studies were funded by the Prevention program of the Netherlands Organization for Health Research and Development (ZonMw prevention 120620024). For the sub-study, we contacted parents who had previously received information about the larger study, and who provided written consent to be contacted by phone or email. Furthermore, since the sub-study involved a onetime one hour interview only, no additional ethics approval was sought.

We aimed to also include parents who had declined participation in the larger prevention study (but did consent to additional contact). Therefore, we tried to keep the inclusion procedures of this sub-study as flexible and low-key as possible: participants were free to determine the method of communication, and were contacted and fully informed by email or phone, accordingly. The provided information included an abstract of the larger prevention study and its aims, and the aims of the sub-study. The topics and approximate duration of the interview were explained, and participants were notified that they would receive 20 euros in coupons for their time and effort. Over the phone, this information was given verbally. Accordingly, verbal (phone) or written (email) informed consent was obtained. Consent was recorded in a document containing an interview transcript, which was actively approved by email by all but one participant (due to technical problems). Data obtained were anonymized and not shared with the therapist of the patients.

### Selection of participants

Parents (patients and partners) who participated in the present study had previously been contacted for participation in a multicenter prevention study, aimed at preventing depressive and anxiety disorders in offspring of depressed and anxious parents, including a screening for additional symptoms and a randomized controlled trial (Nauta et al. 2012). The trial included a 10 session behavioral training for children, with 2 individual parent sessions, aimed at preventing offspring anxiety and depression. The training focused on reducing risk and increasing resilience in offspring, including modules on (1) family functioning and social network, (2) being proud of strengths, (3) positive emotions and events, (4) problem solving, and (5) approach behavior and activation. The therapist addressed each of the modules in the first sessions and then elaborated on the most appropriate module(s) for each child.

Trial inclusion criteria for patients were: treated for unipolar mood disorder or anxiety disorder either currently or in the past five years, with a child aged 8–18 years. Exclusion criteria were: mental retardation, severe alcohol or substance use disorder, schizophrenia or other primary psychotic disorder, schizoaffective disorder, bipolar disorder. Further details on the design of the trial have been described elsewhere (Nauta et al. 2012).

Participants in the qualitative study were recruited from three large mental health institutes. We used purposeful sampling of interview participants that was based on maximum variation, in order to get as many perspectives on the studied phenomenon as possible (Meadows & Morse 2001). In order to attain maximum variation, we interviewed patients and partners, patients with both anxiety and depression, parents of children of different ages, fathers and mothers, and respondents from different institutions. Some parents participated in the prevention study, while others refused to participate. Potential participants were contacted and informed by phone. If willing to participate, informed consent was obtained and an appointment was made for an interview at home or at the psychiatric department.

In qualitative research, the process of data collection and analysis ends when ‘saturation’ is reached (Meadows & Morse 2001). This is the point where no information is added and data replication occurs. Participants were selected via purposeful sampling and recruitment was ceased after saturation (after 24 interviews), resulting in 33 requests to participate with nine declining. Of these nine, two parents did not volunteer any reason; one had already participated in a large national cohort study and the larger prevention study (Nauta et al. 2012); two only stated ‘not interested’. Others provided different reasons like being too busy in general, too busy rebuilding the house, or too busy managing their own anxiety. Others did not want to talk about, or be reminded of their (past) disorder. Eight of these nine parents had also declined participation in the clinical trial.

Table 1 shows the characteristics of the 24 interview participants. Fifteen (62.5%) had refused to participate in the prevention study. Participants were 7 partners and 17 patients. Seventeen of twenty-four (70.8%) were mothers, with children aged 2–26 years (*M* = 11.9). In 2 families both parents suffered from anxiety or depression.

**Table 1:** **Overview of participants**

Subject	Parent	Age	# Children	Partner or patient	Disorder	Participation in prevention study?
1	mother	41	3	patient	Depression (M)	yes
2	father	50	5	patient	Depression (F)	no
3	mother	37	4	patient	Depression (M)	no
4	mother	38	2	patient	Anxiety (M)	no
5	father	48	2	partner	Depression (M)	yes
6	mother	45	2	patient	Anxiety (M)	no
7	mother	50	1	patient	Depression and anxiety (M)	no
8	mother	43	2	patient	Depression (M)	no
9	mother	49	2	partner	Depression (F)	yes
10	mother	48	2	partner	Depression (M), anxiety (F)	no
11	father	58	2	patient	Anxiety and depression (F)	no
12	mother	30	4	patient	Anxiety (M)	yes*
13	mother	41	1	patient	Depression (M)	no
14	mother	39	2	patient	Depression (M)	yes*
15	father	47	2	patient	Depression (M)	no
16	mother	46	1	partner	Depression (M and F)	no
17	mother	42	1	patient	Anxiety (M)	yes*
18	mother	31	1	patient	Depression (M)	no
19	mother	47	1	patient	Depression (M)	yes
20	mother	40	3	patient	Depression (M)	no
21	father and mother	41 (F) 36 (M)	3	patient and partner	Depression (F)	no
22	mother	36	3	partner	Depression (F)	yes
23	father	45	2	patient	Depression (F)	no
24	father	40	2	partner	Depression (M)	yes

*Note*: M = Mother, F = Father. * = participation in prevention study ended prematurely.

### Research team and reflexivity

#### Personal characteristics

The research team consisted of five female psychologists (MN, HF, LM, NB, SE) and three parent interviewers. Each interview was conducted by a duo of one psychologist (LM, SE, NB) and one parent interviewer. Parent interviewers were selected based on their participation in the prevention trial and their interest in the study. Actively including patients as equal partners during interviews had several advantages, such as preventing jargon, establishing trust and recognizing diversity (Abma et al. 2009; Nierse et al. 2012). All three parent interviewers were mothers with secondary education, aged 45–57 years, with 2, 3, or 4 children. Two were patients (with anxiety disorders or depression) and one was a partner of a patient (with depression). All psychologists in the team were MSc or PhD level (health care) psychologists with clinical and research experience.

The team received a two day training by an experienced qualitative researcher (KS) in interviewing: building rapport, empathic listening, probing, and asking open-ended questions in order to make the interviewee feel at ease, decrease social desirability, and deepen the conversation. Furthermore, the team worked together to construct a topic list, and discussed expectations with regard to the answers to the research questions, thus acknowledging the assumptions and biases of the team members.

#### Relationship with participants

A relationship with the interviewee was established prior to the start of the interview. Both psychologists and parent interviewers explained their connection to the prevention trial and the reason for (participating in) conducting the interviews. In the prevention trial, LM had participated as therapist, NB and SE as research assistants.

### Data collection

Qualitative data were collected between June and October 2012 through semi-structured interviews, guided by a topic list with open questions. Interviews and analyses were conducted in Dutch. For the purpose of this paper, the topic list and citations were translated and back translated by a native English speaking professional Dutch-English translator (member of the Society of English Native Speaking Editors), and two of the Dutch authors (MN and HF).

The topic list was based on previous research, the research questions and the research team’s knowledge about and experiences with (non) participating parents in the prevention study (see Additional file 1). The interview schedule was only used to roughly structure conversations, allowing digressing into other topics brought up by the interviewee (Smith 2003). Main questions covered demographic information, and assessed parent’s anxiety or depression, effects upon children, parenting, parents’ help-seeking for themselves and their children, and participation in screening and the randomized controlled prevention trial. In addition, probes and follow-up questions were used to manage and get a better and deeper understanding of the interviewee’s answers (Rubin & Rubin 2011). Interviews took place at the home of the participant or at the psychiatric department. The average duration of an interview was approximately 60 minutes.

### Data analyses

All interviews were, after permission, audio-recorded and fully transcribed (written out line by line) with anonymized names and places. An inductive content analysis was performed in line with the grounded theory methodology (Charmaz 2006). Grounded theory is a systematic methodology for investigating personal experiences. This method involves the discovery of theory through the analysis of data, making it especially suitable for investigating perceptions and experiences without predefined hypotheses, in order to discover new insights or theories.

First, the transcribed interviews were read to identify emerging themes and subthemes. Codes and labels were attached to text parts/citations related to a specific (sub)themes (open coding), leading to a set of descriptive themes per transcript. Each interview was analyzed separately by two individual researchers. Differences regarding emerging labels were discussed and resolved. Then, all labels of all transcripts were compared and redefined, and clustered into themes and subthemes (axial coding). Eventually, overarching main themes were formulated (selective coding), and similarities and differences between cases were identified (cross case analysis of constant comparison) to provide further insight into the research questions.

### Quality procedures

To check the validity, a ‘member check’ (Meadows & Morse 2001) was performed: all interviewees received a summary of the transcript of the interview and were asked whether they agreed with the content. One interviewee did not respond to the member check, all others agreed with the content.

A second member check involved an in depth discussion with two of the three parent interviewers. These interviewers were two mothers (one patient, one partner), and they participated respectively in 6 and 11 interviews. Conclusions from this discussion confirmed the interview analyses and are not reported separately in this paper. Furthermore, ‘check coding’ was used, meaning that three different researchers (HF, MN, KS) were involved in the process of data analysis, in order to enhance the inter-rater reliability (Meadows & Morse 2001).

In qualitative research, the process of data collection and analysis ends when ‘saturation’ is reached (Meadows & Morse 2001). This is the point where no information is added and data replication occurs. Participants were selected via purposeful sampling (see selection of participants) and recruitment was ceased after saturation (after 24 interviews), resulting in 33 requests to participate with nine declining. Saturation was discussed in the research team and reached in this study after 24 interviews.

## Results

The analysis resulted in two main themes per question, with several subthemes, which are discussed below. For an overview, see Table 2.

**Table 2:** **Overview of research questions and main themes**

Research question	Main themes
1. What are parents’ experiences with regard to their own depressive and anxiety disorders and their children’s vulnerability and resilience?	- Impact on Quality of Life (QoL) of the children
	- Parental concerns about the mental health status of children
2. What are parents’ experiences with regard to their own depressive and anxiety disorders and parenting?	- Impact on family QoL
	- Communication about parental illness
3. What are parents’ experiences with help for their children and is there a need for help (e.g. preventive interventions)?	- Lack of focus on children in parental treatment
	- Parental perspectives on the need for professional help for children
4. What are parents’ reasons for (not) participating in a prevention study with their children?	- Reasons for not participating: parental overburden, child burden, child refuses to participate, stigma, shame, no worry about children
	- Reasons for participating: need for prevention, helping others, importance of research, child likes to participate
5. What are parents’ experiences and advice with regard to participation in a prevention study?	- Positive experiences: personal information from therapist, ‘depth’ in conversations with offspring
	- Negative experiences: too many measurements and questionnaires

### Offspring vulnerability and resilience in relation to parent depressive and anxiety disorders

Related to the first research question, two key themes emerged: *Impact on the Quality of Life (QoL) of the children*, and *Parents’ concerns about the mental health status of children*.

#### Impact on the Quality of Life (QoL) of the children

Parents’ perceptions on the relationship between their own or partner’s disorder and their children greatly differed. Most parents believed their problems did not influence offspring QoL, and that parents can keep that part of their life away from their children. Especially fathers who were patients seemed to believe that children are not affected by parental disorder.

#5 *“In theory, neither of them had any trouble because of it [father’s depression]. Life just went on as usual, in principle… Kids are just kids, they quickly forget that something is going on…No, the kids didn’t suffer at all.”*

Only few parents did noticed that children sensed it when parents were not feeling well, and thereby realized that their disorder did impact their children.

#3 *“The children have emotional antennae. If I had a bad day, they sensed that immediately.”*

Although parents tried to avoid negatively affecting their children’s QoL in order not to burden/encumber them with responsibilities, parents sometimes did notice that their children were more caring and tried to take their parents into consideration.

#11 *“Children have an enormous need to rescue their parents. So I think that she really sensed that there was tension. And tension under the surface is more cruel than tension that comes out, you know. So then she was incredibly busy trying to do the best she can [for me].”*

#### Parents’ concerns about the mental health status of children

While most parents believe their disorder does not really impact the QoL of their offspring, almost all parents worried about their offspring. Parents noticed that their children were more sensitive, anxious, sad, emotional, cry more often, are easily upset, insubordinate or have trouble in school. However, even though parents acknowledged the symptomatology in their children, they did not explicitly seem to make a direct link between their own mental health problems and their potential impact on child symptoms. One mother (#21) described her daughter as being “*a little bit unsure, afraid of making mistakes, needing reassurance”*. Another parent remarked:

#18 *“He seriously felt that if something didn’t work then he was stupid… Then he could be really down on himself. Like ‘I can’t do it’ or ‘whatever, It’ll never work for me’. And tearful, he was that too. Really emotional…”*

A lot of parents furthermore recognize their own or their partners’ anxious and depressive symptoms in their children, as illustrated by the next quote:

#12 *“I notice that she [daughter] panics quickly. For a while she’s really been saying [impersonates daughter]: ‘Oh! I’ve got so much pain here’ and then hyperventilates and panics. I think, “Ooh, that’s how it began with me…’ So I recognize it really well, and I think, ‘Ooh, I must keep her calm, I must take care that she doesn’t get further sucked into this’.”*

### Parenting and parental depressive and anxiety disorders

The second research question about parents’ perspectives on the influence of their disorder on parenting revealed two key themes: *Impact on the family Quality of Life (FQoL)* and *Communication.*

#### Impact on the family Quality of Life (QoL)

According to the participants, parental anxiety and depression can influence family functioning and family QoL in different ways. Most parents were in doubt regarding the influence of their mental disorder on family QoL, and tried their best to parent as ‘neutrally’ as possible. However, parents also realized that the impact of parenting and the impact of parent psychopathology on family QoL can be two different things:

#23 *“I think their upbringing wasn’t really different, I mean, that’s what we aimed for at least, to be as neutral as possible. But well, of course they noticed something, probably, for sure they will have noticed more than they are aware of.”*

However, most parents also reported a more negative atmosphere in the acute phase of an anxiety disorder or depressive episode. A partner of a father with a depressive disorder remembered that in the acute phase of the disorder, her husband’s depression influenced the atmosphere at home:

#9 *“[Father] was saying like, ‘You don’t have any problems because of me’. But it was so obviously present, the elephant in the room.”*

Independent of parents’ perceptions about the impact of their disorder on the well-being/quality of life of the children, almost all parents realized that their children notice some of their depressive or anxiety symptoms, such as fatigue, withdrawal, irritability, sadness, and anxiety. These symptoms may influence the QoL of the family (see Table 3).

**Table 3:** **Parental symptoms influencing family Quality of Life**

Symptoms	***Citations***
Fatigue	*#10 “I was in bed a lot … Then they came home from school and their mum was in bed again, so that wasn’t very nice. Because I was just so very tired, above all.”*
	*#4 “The only thing she (daughter) said was: ‘Mum is in bed more often’.”*
Withdrawal	*#9 “… That he (father) didn’t come along and was always sitting on the couch and didn’t want anything… They (children) do find that very annoying.”*
Irritability	*#24 “then he [father] doesn’t feel well, then he doesn’t feel happy and then he’s simply more sensitive and then he’s quicker angry at the children while he normally isn’t quick to be angry at the kids.”*
	*#19 “Well, conflict more than anything. I was easily irritated. As much by my partner and as by my kid. Luckily, I reacted as little as possible to my kid. She can’t do anything about it, but unfortunately she still got some of it. Well, you can’t avoid that. You want to but it doesn’t work.”*
Sadness	*#10 “We laugh too little. It is a sort of serious family.”*
Anxiety	*#17 “Like the other day, there was another disco at school, and well, then I start calling. That’s how she (daughter) notices. Then she missed like a hundred calls and they’re all mine. And then she thinks like ‘damn I should ‘ve called my mother.”*

Some patients and partners realize that depression or anxiety related symptoms influence a parent’s parenting style.

#14 *“A shorter fuse. In general I feel like, what does it matter, let them go and have fun. But when it is too much, it’s TOO MUCH, and then I go off the deep end, and then later I’m crying, like ‘what did I do’… and then I think ‘those poor girls they can’t help it either. They just want attention.”*

Sometimes, parents argue a lot.

#10 *“They were afraid that we would separate, because (father) and I fought a lot.”*

Furthermore, a lot of parents remarked that the parent with anxiety of depression withdraws from family life, leaving the partner and sometimes grandparents, other family members or neighbors in charge of the family.

#18 *“All I was doing was sleeping. So my mother took over at a certain point. My twin sister also came to take care of him too, because I wasn’t leaving the house. I wasn’t really there…so they took over everything. They cooked, they did the housework, they really did everything. …I didn’t really see him [son] in that period.”*

Also, during an anxiety or depressive episode, house rules were sometimes omitted, because parents with anxiety or depression have greater difficulty with maintaining order.

#3 *“Sometimes saying ‘yes’ when you really want to say ‘no’ just so you don’t have to deal with the struggle, that is just easy.”*

Interviewees explained that because of their anxiety or depression, they are more prone to protect their children and to keep in control than other parents, being afraid that bad things may happen to them, or that they may develop a mental disorder. A mother described how her anxiety disorder influenced her parenting style and caused her to worry more about her daughter:

#17 *“…in the beginning I took her with me everywhere. She wasn’t allowed to go anywhere alone, but now she’s older and she wants to be away from me more frequently… noooo, no way.”*

#### Communication about parental mental illness

Parents had very different opinions with regard to informing their children about their mental disorder. Parents stressed the importance of keeping their problems out of their children’s lives and parents seem to value secrecy with regard to their mental health problems. However, they do not always succeed.

*#12 “I always keep it well hidden, since I always go into the kitchen… but there were sometimes periods that it got really bad and I was so panicked…that I became short-tempered and said ‘Leave me alone’ […] But naturally they didn’t understand because everything was fine […] and then, suddenly, I wasn’t at all fine anymore.”*

Some parents explained that they talk as little as possible about their complaints, in order not to burden their children. Also, parents believed it is best not to talk about it with their children, because they think (young) children will not understand.

#14 *“I think they’re too young, I don’t want to burden them with it.”*

A lot of parents did tell their child that ‘something’ is going on, but do not elaborate. Anxiety and depression are not openly discussed, but explained to the children in terms of headaches, being tired or ‘can’t take much’. On the other hand, there were also a few parents who did tell their children about their anxiety or depression and were more open about the subject of mental disorders. These parents explained that they like to be open and honest with their children and do not want to lie.

#15 *“I call it gloominess (…) Compared to what I knew in my childhood, they know that for pain in your knee or your stomach you go to a white-coated doctor, but that there are also doctors for thoughts. For if something awful has happened or you can’t get over something or if it continues too long. I find that really positive.”*

Often parents started explaining at greater length when the children got older, when children started asking questions themselves, or when minimal information led to confusion.

#9 *“…we had said that ‘Daddy’s sick in his head’, and then they saw things on TV, you know, about Norway*^*a*^*…And there it was said, ‘that man is sick in his head’, so then we went and made it more specific, ‘there are different sicknesses you can have in your head’. So we did make it clear that it wasn’t the kind of sickness that that man [on the TV] had, and he had more going on than that, but, well, it was hard to explain.”*

#15 *“Once they came home being very emotional, because children in their class asked them what was wrong with me… Because I was in the hospital. It was something with my brain… Then it turned into a brain tumor and well you can imagine how that conversation turned wrong. (…) A brain tumor means you’re almost dead. So they came home very frightened.”*

It appeared to be a dilemma for parents what to tell their children. Some parents are inclined to a one-sided approach; i.e. you can either say nothing or way too much.

#11 *“I am very reserved [in what I tell my children]. (…) my mum had the same as I have, and she once called me to tell me that she would throw herself in front of a train and I always keep that in mind. That, I will never tell my kids. ”*

For some parents, having to talk to their children about their disorder was a reason not to participate (also see Table 5). Parents were ashamed and afraid of what their children might think when they would discuss the parent’s mental health problems.

#8 *“I think I am secretly really afraid of that, of what she [daughter] might say.”*

### Parental perceptions on the need for professional help for children (e.g., screening and preventive interventions)

The third research question was about the need for screening or (preventive) help for children with parents with anxiety or depression, with two emerging key themes: *Lack of focus on children in parental treatment* and *Parental perspectives on professional help for their children*.

#### Lack of focus on children in parental treatment

Parental experiences with treatment and mental health care were discussed with regard to focus on offspring. Most parents said that the treatment they received was primarily focused on themselves, treating their anxiety disorder or depression.

#18 *“[Treatment] was all about structure, like ‘what are you going to do today’, that’s it really. You just talk about the rest of the day and what you did, and what you did the day before and what you could have done differently. So my son wasn’t really involved (…) Also, It was only about you, so it wasn’t like ‘if you have children or a partner..’ … no.”*

Parental treatment is regarded as positive, by both patients and partners. Plus, treatment can also be helpful for the rest of the family. Some parents used parts of their treatment to explain things to their children or notice that treatment helps them to talk more easily about their emotions.

#23 *“Things I also apply while parenting, when we talk about things, like ‘you think that, but is it realistic?’ I mean, I ask them that too. But then without saying ‘this is therapy’…”*

In one example, the adolescent child was an important part of parent’s exposure therapy:

#17 *“…that’s why there’s a lot said about letting go. That you do it step by step…that’s how you do it. Little baby steps and then let go, since she needs to develop into a young lady soon. And if we don’t do that, she’ll be here too, later. Yeah, I know it. That’s why I’m here too. To let her go. But, how do I do that? I find it terrifying.”*

To conclude, when parents were in treatment, little to no attention was paid to offspring, family environment, and parenting skills. However, most parents indicated that they did not miss this subject per se.

#18 *“That wasn’t on my mind at all at that moment, so I didn’t miss it or anything. I didn’t think they should have included that, no.”*

Furthermore, during treatment and in general during the acute phase of the disorder, patients tended to be so preoccupied, that also paying attention to offspring or family seems too much to handle.

#12 *“Maybe afterwards, when it (treatment) really was at its end, maybe then we could have focused on the children …”*

Also, some parents noted that their children were doing well, and therefore expressed no need for focus on children, or screening.

#13 *[When asked about treatment or screening for child] “Well, not for her (daughter), no, no. That is really not necessary, she was obviously doing fine.”*

#### Parental perspectives on the need for professional help for their children

While parents in general recognize the importance of prevention for offspring, a lot of parents found it difficult to articulate what kind of help they would want. Parents thought they would have gotten help if they would have known what to ask for.

#19 *“You yourself are like ‘how do I do this, how can I do that?’ and at that time, well you have health care professionals close by, but still, you’re missing something. You just can’t point out what it is, at that moment. Yeah, that’s difficult.”*

However, more than half of all interviewees did articulate a need for focus on the children. More specifically, there was a need for (preventive) screening and (easily accessible) parenting support. Furthermore, parents communicated a need for practical home support and help with communicating their mental health problems to their offspring, see Table 4.

**Table 4:** **Parental perspective on professional help for offspring**

Parental perspectives	***Citations***
‘Children’ as a topic in regular mental health treatment	*#10 “…I think it’s really important that the Mental Health Care Center pays attention to that, that people who have kids, that attention is paid to them, over how do you handle that.”*
Practical support	*#13 “I needed most that she (daughter) just had a normal and fun life, that she has enough diversions and does fun things. It’s important that, now and then, someone else takes over.”*
Parenting support	*#1 “…if I told where I struggled (with regard to parenting) then they usually said that ‘those are parenting problems that everyone comes up against’. I understand that, but I still think that some aspects can be identified where more support can be offered.”*
Family psycho-education	*#10 “I think it is wise that people who have this [anxiety or depression], that they are taught how to deal with it, when you have a family. And that you get some instructions like ‘how do I tell my kids’ and ‘how do I deal with this so that they better understand what is going on with their mum and what do you tell them and what don’t you? I find that very difficult.”*
Help with screening for child symptoms	*#15 “Please check on them and tell us if everything is normal. It’s like my compass isn’t working and I can’t sail. And my partner finds it all really difficult. And she is really unsure about how to raise them… if we’re doing okay overall. I don’t feel that way, but she does. So she’s all alone in her uncertainty at the moment.”*
Preventive child intervention	*#1 “…that she doesn’t suffer the same consequences as I did, since that was a hard way of learning. If she can get a better grasp now, that would be great.”*

Furthermore, partners in particular seem to plead for support for the family. Partners suggest a combination of practical family support, psycho-education and support for partners and support for offspring. For example, speaking about her partner, who was the patient, one mother remarked:

#9 *“…that’s what I really missed by the Mental Health Care Center, when (father) went there, like ‘Hello! There’s a family as well.’ There are kids there and (father) told you that it was going badly with him, but then what happens to us? (…) I had no idea, only lots of questions, that we couldn’t do anything with.”*

Three participating parents indicated that they did not experience any need for professional help for the children before receiving information about the preventive trial. However, the provided information changed their opinion and made them recognize the value of screening and preventive intervention for their children.

#12 *“That [the question for participation] came from my psychologist (…) And I thought: ‘That is a good thing’, you know, because of course I do recognize things and I think, it is good to see how she (daughter) is doing.”*

In many families, one or more children were in treatment. Parents described a divergent pattern of problems: ADHD, behavioral problems, emotional problems, autism, mental retardation, anxiety disorders, depressive disorders, suicidality, eating disorders. Treatment for these problems varied from school support or primary care, to psychiatric family admission and compulsory admission in a psychiatric hospital. Parents are prone to seek help when a child is suffering. However, participants report that children’s symptoms are hard to recognize for parents unless they reach a severe level. Parents commented that they only sought help when something was really going wrong.

#11 *“It [eating disorder symptoms] was lying under the surface, so only when it became really heavy, when the youngest had to be hospitalized, because she was near death. And the oldest had swallowed too many pills [iron tablets]. Yes, then something could be done.”*

#20 *“She frequently didn’t feel good about herself then, we’d all sensed that, but not that it was so deep. One day we were walking toward the caravan and she started to cry and said, ‘Mama, I really want to die. I don’t want to live anymore. I feel so miserable and I don’t want to feel that way anymore.’ At that moment I thought, ‘Huh, what?’ We really hadn’t seen it coming, totally not. At the moment we thought, ‘Is this a one-off thing?’ And we got talking and she was sure that she wanted to be dead, that she wanted to kill herself, but she didn’t know how yet. It was already that far along. We really didn’t see that coming.”*

### Participation in a prevention study

Reasons for (not) participating in screening and a prevention study with your children are extensive. Table 5 summarizes barriers for participation, whereas Table 6 summarizes reasons for parents to participate.

**Table 5:** **Reasons for not participating in a prevention study**

Main themes	***Citations***
Parental overburden	*#3 “I can’t do this [participating] as well”*
- Parental symptoms and disorders	*#7 “I would have liked to [participate], but then again not really, and I think ‘how do I get there? I am afraid to drive with these meds”*
- Ending the Mental Health care period in your life	*#2 “…and if you’re almost finished at a particular moment, then I want to finish up too… I don’t think that you should, if you are finished…that you should keep dragging things up by participating in all sorts of studies, because then it keeps it alive and you can never finish. And now I’ve finished this off. Done”*
- Time investment and paperwork	*#4 “It’s purely the time investment, (…) And you just shouldn’t give me a pile of paperwork… [laughs]. Filling out paperwork, that’s a horror for me. I also outsourced all paperwork at home”*
	*#21 “Also, I work 40, 44 hours a week, so… when I come home… then there are 2 nights that my wife works, so the time you have left.. plus she also works one day, add up sports one time a week (…) we don’t have time left”*
- Participation too confronting	*#22 “I could imagine that people would not participate, because they will be confronted with their own issues. For example: ‘do I spend enough time with my children and do we have enough fun?’ Things like that, do I dare to hold up a mirror? Their father finds that very difficult, so he didn’t participate”*
Child burden	
- Protecting children from possible negative effects of participation in preventive research	*#23 “I have my doubts that it’s completely without risk…I call that a reversed placebo effect…if you treat people then they think, ‘I am being treated thus I am sick, so there is something going on’. And, I’m not waiting on that, naturally… that would be my fear“*
	*#14 “I just didn’t want to make her worried”*
- Child too young	*#23 “When they’re older it becomes easier for them to put it in context? Then they can participate themselves, choose for themselves if they want to participate, but, to be honest, we still have to prepare them for it… But when they’re young it’s just different, and if you don’t see any problems, you don’t go looking for them”*
Children refuse to participate	*#16 “It was, naturally, on a voluntary basis, so (son, almost 14) is naturally really an adolescent who won’t do things in his free time, which is sacred to him”*
Stigma	*#14 “He [father] says: ‘[if we participate] then she’ll get a patient file, and she gets labelled and she’ll never get rid of that label’*
	*#17 “…I don’t want anything to do with the [Mental Health] business”*
Shame, embarrassment	*#23 “The reason for me for not participating was that we should have told them [children] the whole story, and well, it’s a difficult story…”*
	*#8 “I think I am secretly really afraid of that, of what she [daughter] might say”*
No worries about the children	*#23 “And I don’t see that they have a problem,… I'm actually 99.9 percent sure, symptom-free, which is good”*

**Table 6:** **Reasons for participating in a prevention study**

Main themes	***Citations***
Prevention	
- Parents recognize their own depression/anxiety in offspring	*#24 “Seeing that we did recognize those things in him [son, 11]. Before this [participation in prevention study] we already thought, let’s take him to the doctor. ‘Could it be that childhood depression, or the like, exists?’”*
- Help, support for parent and child	*#19 "If she learns to be more resilient etc., that can only be good"*
	*#1 “Now she lets us know…she can’t stop herself going over things in her head, ‘how can I stop this?’… I wanted support for her so badly and that’s why we signed up”*
- Prevention of child problems	*#15 “I want to have them checked preventively. I want someone to tell me that everything is still normal, given the circumstances, or that they need treatment themselves in whatever way”*
Helping others	*#19 “Those were the most important reasons. Yeah, I think, when I tell my story and it helps other people who suffer encounter the same problems, then …”*
Research is important	*#10 “We both were researchers, so you know… it’s.. hard to collect data – I call it data, we studied other things – … it is very important that research on this topic is conducted”*
Child likes to participate	*#9 “My son found it very interesting, and he wanted to participate really badly”*

Parental overburden was an important reason for parents not to participate in preventive research with their children. Parents indicated not having the time nor the energy to participate. Factors related to parental overburden were parental symptoms, the need to end the ‘mental health care period’ in their life, the required time investment and paperwork associated with participating in a prevention study for offspring, and the fact that participation was experienced as confronting.

Other reasons for parents not to participate were related to child burden. Parents wanted to protect the children from possible negative effects of participation, or found their children too young to know about their parents’ illness, and to participate in research. Parents with children aged 8–12 discussed that they might participate in preventive research when the children are older. Parents reckoned that older children might be more aware of their own and others’ emotions, might be better able to talk about them, and might furthermore be better able to make their own decisions with regard to participation to research and intervention. However, in families with children aged 12 and older, an important reason for not participating was that these adolescents were not interested and refused to participate. Furthermore, stigma, shame and embarrassment about anxiety and depression or being in treatment were reasons for parents for not participating. Finally, some parents mentioned no worries about the children as a reason for not participating in screening and prevention.

Parents who acknowledged the need for prevention were motivated to participate in the study. Recognition of their own symptoms or disorder in their offspring, the need for support for themselves and their children, and prevention of child problems were reasons for parents to participate. Also, helping others by participating, and knowledge about scientific research were mentioned by parents as reasons for participating. Finally, some parents discussed participation with their children, and children themselves were interested and motivated to participate.

### Parental experiences and advice with regard to participating in a prevention study

Eight out of twenty-four interviewed participants participated with their children in the prevention study. The fifth research question asked participants about participants positive and negative experience with the study (Nauta et al. 2012), with regard to the screening, the measurements, the randomized controlled trial, and the training (for participants who were randomized in the training condition of the prevention study).

#### Positive experience

As an important factor in their willingness to cooperate in research, patients mentioned the personal information they received face to face from their therapist regarding the study.

#4 *“Like [therapist] did it, just asking me like ‘would you like to participate’. I think that works very well.”*

An advantage of participation parents mentioned was the fact that filling out questionnaires during measurements and a diary during the intervention gave room for a conversation about topics like emotions, anxiety and depression. Therefore, parents experienced more ‘*depth*’ in their conversation with the children.

#22 *“And I really liked the diary, we filled it in every week. I liked that a lot, because I said to [son] ‘you were quite angry the last few days’ and then there was an opportunity to say ‘why were you angry?”*

#### Negative experience

Comments of parents on participating in the prevention study were primarily related to the questionnaires in the study: there were too many measurement points, the amount of questions was too much, and the questionnaires contained difficult and negatively formulated questions.

#13 *“Too much of a hassle. And all those questionnaires looked alike (…) if you’re the one who has to fill them in you think like ‘another one??’. Very monotonous… and for [daughter] I found them too much about negative mood. (…) She felt really down afterwards….”*

## Discussion

The purpose of this study was to obtain qualitative data on parents’ perspectives on parental anxiety and depression, parenting, offspring risk, and the need for and barriers to parent participation in offspring preventive health care, offspring screening and randomized controlled trials (in order to shed some light on the low participation rates of families in offspring prevention). We conducted 24 interviews with parents (both patients and partners, fathers and mothers). Even though parental perceptions and experiences regarding offspring risk, vulnerability, communication and the need for (and the importance of) preventive intervention varied widely, some general themes emerged, as summarized below.

1. Impact on offspring: no perception of a direct link between parent symptoms and offspring Quality of Life, but concerns about children’s symptomatology.
  Parental perceptions on offspring risk greatly varied. Most parents believed their problems did not influence offspring QoL. Especially fathers seemed to believe that their children are not affected by parental disorder. In line with previous research (Boyd et al. 2006), parents did not seem to make a direct link between their depressive and anxious symptoms and the potential impact on offspring QoL. However, almost all parents did articulate concerns about their children’s mental health status. Previous qualitative studies never explicitly asked parents about the impact of parental illness on their offspring, but child problems are frequently mentioned by parents (e.g., depression, ADHD, drug abuse, learning disabilities, significant levels of emotional and behavioral problems (Boyd et al. 2006; Stallard et al. 2004). Whether parents attribute the development of child problems to their own mental illness and genetic transmission of risk (nature) or to environmental factors, such as parenting (nurture) has not been explicitly verified in this sample.
  Parents barely mentioned offspring resilience and protective factors in relation to parental anxiety and depression. A study, aimed at screening offspring for high risk and offering a preventive training which included preventive activities directed at resilience (Nauta et al. 2012) may therefore have been a step too far, since parents should first be informed on offspring resilience in relation to the transmission of risk.
2. Impact on parenting: parent mental illness does influence family Quality of Life, while parents value secrecy to protect the children.
  Parents described that parental mental health problems influenced family Quality of Life. Children notice parents’ fatigue, withdrawal, irritability, sadness, and anxiety. Furthermore, anxiety and depressive symptoms influence a parent’s parenting style (e.g., a lack of patience, withdrawal from care, inconsistent parenting, and overprotectiveness). These parenting difficulties are in line with previous research on mothers with depression (Boyd et al. 2006). In quantitative research, it has also been well established that parental anxiety and depression are likely to affect the quality of parenting (Downey & Coyne 1990; Goodman & Gotlib 2002; Lieb et al. 2000; Moore et al. 2004). However, parents did not seem to relate these parenting issues or FQoL to offspring mental health.
  A second key theme regarding parental disorders and parenting was communication. Parents reported various reasons for secrecy about their mental disorder. Most parents were hesitant to provide any information to their offspring or only provided minimal information when necessary (e.g., ‘headache’, ‘sick in the head’). Unlike privacy, which in the West is considered a healthy characteristic of the autonomous adult, secrets are often troublesome, creating distorted perceptions and strained relationships (Imber-Black 1993). In line with previous research (Stallard et al. 2004), however, parents in the current study were concerned that the provision of information may have a negative effect in terms of unnecessarily burdening the child with details of the parent’s illness. Furthermore, parents believed young children would not understand. Often, additional information was provided when minimal information led to confusion and worries in children. Only a few of the parents knew how to explain their anxiety or depressive disorder to their offspring in an open and age-appropriate way. Previous research on offspring of depressed parents has emphasized the importance of informing children about parental mental illness (i.e. making offspring understand what anxiety or depression means, making them realize that they are not to blame), and the possible adverse effects of lack of information (Beardslee & Podorefsky 1988; Focht-Birkerts & Beardslee 2000). However, according to parents, this information is currently not provided in adult mental health care.
3. Professional help for offspring: wish for psycho-education, symptom screening in offspring, and referral for real problems rather than intensive preventive interventions.
  Parents viewed mental health treatment for themselves as helpful and beneficial. However, in contrast with the current guidelines regarding youth depression (National Institute for Health and Care Excellence 2005), little or no attention is paid to offspring or parenting in parental treatment, according to the parents in our study. Parents did not consider this to be unfavorable and indicated that in the acute phase of an anxiety disorder or depressive episode, it is important to focus treatment on parental symptoms and disorders. This is in line with findings from a large longitudinal mother-child study (STAR*D) which emphasized that effectively treating parental depression is associated with decreased psychiatric symptoms and improved functioning in offspring (Pilowsky et al. 2008; Wickramaratne et al. 2011). Informing parents of this advantage of parental treatment should be included at the outset of parental psycho-education in adult mental health care.
  In most families, offspring intervention only became a priority when children developed symptoms, and parents expressed a need for discussing child-related concerns with their therapist. Parental needs for offspring-related interventions mainly consisted of help with screening for symptoms. In addition, parents expressed interest in prevention, parenting support, practical support, and family psycho-education, without directly including (i.e., ‘burdening’) the child. However, most parents emphasized that they find it hard to articulate their own and their children’s needs.
  It is not surprising that recent randomized controlled trials intending to investigate preventive interventions for offspring have struggled to include enough participants to achieve sufficient power. It appears that offspring-focused prevention does not fulfill parents’ needs for help. Parents suggest a more parent-focused approach including psycho-education and parenting support. Offering a parenting program in adult mental health care that meets parental needs and preferences is a potential successful strategy for preventing problems in children. Furthermore, offering such a program in groups could have the advantage of peer-to-peer support.
  Parents indicate that little to no parent support is currently provided in adult mental health care. Barriers that currently may prevent an effective consideration of offspring needs in adult mental health may include time pressure, and a lack of child-focused skills in adult mental health (Stallard et al. 2004). Therefore, professionals’ opinions regarding implementing parenting-related interventions in adult mental health care should be investigated.
4. Barriers to participation in research: parental overburden and perceived lack of necessity for child intervention.
  The findings reported above show that important reasons for not participating in research are parental overburden (including parental disorders and additional demands of the study), perceived lack of necessity of professional help for offspring, worry about negative effect on offspring, shame and stigma, and children refusing to participate. This is in line with previous studies confirming that the additional demands of a study may cause concern for some parents, and influence their decision to participate (Ross et al. 1999). In line with studies of parents with a variety of mental problems (Stallard et al. 2004), parent’s needs seem to overwhelm and obscure the needs of offspring, and parents did not wish to involve their children, fearing that participation would cause them further distress.
  In contrast with the barriers to participation, some of the most commonly-mentioned motivators to participation were worry about the children’s symptomatology, and the need for support, and prevention. Furthermore, helping others, the importance of research, and children wanting to participate were named as reasons for parents to participate in the prevention study.
5. Positive and negative experiences with participation in a prevention study.
  Parents participating in the prevention study (Nauta et al. 2012) were positive about participation. Interestingly, when a therapist had played a prominent role in informing parents in the inclusion phase, and parents experienced personal communication, parents were more willing to participate. The therapist recruiting for the trial has been reported to have the greatest influence on the decision to enter the trial (Ross et al. 1999).
  Negative feedback included the ‘pile of information’ provided, questionnaires, and the negative content of questionnaires. Previous research investigating multiple randomized controlled trials and barriers to participation also indicated that recruitment information should be simply presented in order to encourage participants to participate in research (Ross et al. 1999). Furthermore, shortening forms by removing unnecessary standardized content improves understanding (Flory & Emanuel 2004; Rogers et al. 1998).
  The inclusion of patient research partners in our qualitative study is in line with the emerging trend of patient participation in psychiatric research (Baart & Abma 2011). Including patients’ perceptions and needs may improve the quality of randomized controlled trials. Langston et al. (Langston et al. 2005) reported how a multi-centred randomized controlled trial, in which patients were involved, led to higher quality of trial information, well-targeted sharing of research findings and well-informed and motivated participants.

### Strengths and limitations

Major strengths of this study are the inclusion not only of patients and mothers, but also partners and fathers, and using purposeful sampling rather than convenience sampling. Furthermore, the data represented here are based on participant’s responses to open-ended questions collected using semi-structured interview schedules, which increases the validity of the findings. Another important strength of this study is the enquiry of parent perspectives and barriers in a group of parents that did or did not participate in an prevention study. Therefore, the barriers mentioned were not hypothetical, but rather direct experience. Furthermore, to obtain these perspectives and experiences, interviews were conducted by psychologists, aided by trained parent interviewers who resembled the group of interest. This strengthened our results by building rapport during the interview, establishing trust and preventing jargon and socially desirable answering (Abma et al. 2009; Nierse et al. 2012).

However, this study also has a number of limitations. First of all, this sample consisted of parents who agreed to be interviewed about their or their partner’s anxiety or depressive disorder, offspring and parenting, suggesting that these parents had insight into their illness and valued their parental roles. Thus, this sample might not represent parents who are less inclined to talk about caring for their children. In addition, the sample size of our study was relatively small compared to a quantitative study. However, in grounded theory, sample size is determined by data saturation and theoretical completeness (Charmaz 2006), which can already be achieved after 12 interviews (Guest et al. 2006). Finally, although the perceptions of parents give interesting input to develop preventive activities, these views may not necessarily be best to follow. Wishes are different from effective strategies. Only a controlled evaluation of the efficacy of any newly developed strategy should be the basis of any subsequent changes in policy or intervention.

## Conclusions

Patients in adult mental health care should be given the opportunity to attend parent psycho-education groups, where knowledge of impact of disorders on child and family QoL and parent–child communication is strengthened, and guilt- and shame-related topics are tackled. A second step can be to provide opportunities for screening on child symptoms and functioning, and referral to youth mental health centers if necessary. Such an infrastructure may also facilitate participation in research trials, by increasing parental knowledge, decreasing guilt and shame, and changing to less “burdening” preventive interventions.

Future studies should then investigate the efficacy of these proposed interventions. Furthermore, mental health care professionals’ opinions on parent-related interventions should be investigated in order to effectively implement a more parent-focused approach in adult mental health care. Last, in order to pave the way for a discussion about offspring in adult mental health care, it is important that information about offspring is added in the guidelines for mental health care professionals working with adults. While current guidelines for health care professionals in child and youth psychiatry recommend that offspring of depressed parents should be referred for depression assessment (National Institute for Health and Care Excellence 2005), information on offspring is currently lacking in guidelines on the treatment of mental health problems in adults (e.g., National Institute for Health and Care Excellence 2009).

### Endnote

^a^The Norway attack in 2011: A man opened fire at the participants of the Labour Party’s youth camp, killing 69 of them.

## Electronic supplementary material

Additional file 1: **Topic list.** (DOCX 15 KB)
