# Supplementary material for: Parents’ perceptions on offspring risk and prevention of anxiety and depression: a qualitative study
Source: BMC Psychol. 2014 Jun 30;2(1):17. doi: 10.1186/2050-7283-2-17 (PMC4363459; doi:10.1186/2050-7283-2-17)
Supplement: Supplementary file 1 — Additional file 1: Topic list. (DOCX 15 KB) [file 40359_2014_41_MOESM1_ESM.docx]

**Additional file 1 – Topic list**

1. Demographic and clinical information

2. Parental perceptions on parental depression / anxiety and children

3. Parental perceptions on parental depression / anxiety and parenting

4. Should mental health services focus on children of patients? And how? Would you

participate to screening or training for offspring?

5. What are reasons for participation in research on screening and preventive treatment?

What were reasons for you to participate or not to participate?

6. STERK-study information package: what is your impression of the information material?

What could be improved?

7. Conclusion and ending conversation
